# Evaluating staging laparoscopy indications for pancreatic cancer based on resectability classification and treatment strategies for patients with positive peritoneal washing cytology

**DOI:** 10.1002/ags3.12719

**Published:** 2023-07-18

**Authors:** Takamichi Igarashi, Mina Fukasawa, Toru Watanabe, Nana Kimura, Ayaka Itoh, Haruyoshi Tanaka, Kazuto Shibuya, Isaku Yoshioka, Kenichi Hirabayashi, Tsutomu Fujii

**Affiliations:** ^1^ Department of Surgery and Science, Faculty of Medicine Academic Assembly, University of Toyama Toyama Japan; ^2^ Department of Diagnostic Pathology, Faculty of Medicine Academic Assembly, University of Toyama Toyama Japan

**Keywords:** pancreatic cancer, peritoneal washing cytology, staging laparoscopy

## Abstract

**Introduction:**

The prognosis of pancreatic ductal adenocarcinoma (PDAC) in patients with positive peritoneal washing cytology (CY1) is poor. We aimed to evaluate the results of staging laparoscopy (SL) and treatment efficacy in CY1 patients based on a resectability classification.

**Methods:**

We retrospectively reviewed 250 patients with PDAC who underwent SL before the initial treatment between 2017 and 2023 at the University of Toyama.

**Results:**

The breakdown of cases by resectability classification was resectable (R):borderline resectable (BR):unresectable locally advanced (UR‐LA) = 131:48:71 cases. The frequency of CY1 increased in proportion to the degree of local progression (R:BR:UR‐LA = 20:23:34%), but the frequencies of liver metastasis or peritoneal dissemination were comparable (R:BR:UR‐LA = 6.9:6.3:8.5%). Most CY1 patients received gemcitabine along with nab‐paclitaxel therapy. The CY‐negative conversion rates (R:BR:UR‐LA = 70:64:52%) and conversion surgery rates (R:BR:UR‐LA = 40:27:9%) were inversely proportional to the degree of local progression.

Comparing H0P0CY1 factors for each classification, patients with H0P0CY1 had significantly more pancreatic body or tail carcinoma and tumor size ≥32 mm in R patients, whereas in BR patients, duke pancreatic monoclonal antigen type 2 (DUPAN‐2) ≥ 230 U/mL was a significant factor. In contrast, no significant factors were observed in UR‐LA patients.

**Conclusion:**

The CY1 rates, CY‐negative conversion rates, and conversion surgery rates varied according to local progression. In the case of R and BR, SL could be considered in patients with pancreatic body or tail carcinoma, large tumor size, or high DUPAN‐2 level. In UR‐LA, SL might be considered for all patients.

## Graphical abstract

The CY‐positive rates, CY‐negative conversion rates, and conversion surgery rates varied according to local progression. In the case of R and BR‐PDAC, staging laparoscopy could be considered in patients with pancreatic body or tail carcinoma, large tumor size, or high DUPAN‐2 level. In UR‐LA, staging laparoscopy might be considered for all patients.

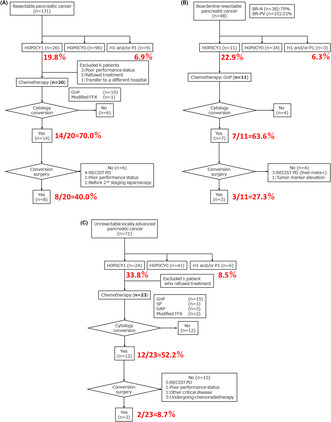

## 1 INTRODUCTION

Pancreatic ductal adenocarcinoma (PDAC) is one of the carcinomas with the poorest prognosis, with a 5‐year survival rate of <10%.^1^ The prognoses of patients with pancreatic cancer and positive peritoneal washing cytology (CY1) are significantly poorer than those with negative cytology (CY0).^2^, ^3^, ^4^ In the current Japanese guidelines for treating pancreatic cancer (7th edition), CY1 is not treated as distant metastasis. However, the clinical practice guidelines for peritoneal malignancy 2021 suggest that pancreatectomy should not be performed for CY1.^5^ In addition, the clinical practice guidelines for pancreatic cancer 2022 from the Japan Pancreas Society states that upfront surgery for CY1 pancreatic cancer is not recommended.^6^ Thus, the treatment strategy for CY1 patients remains controversial. In our department, we consider CY0 as a prerequisite for radical surgery, and we perform staging laparoscopy (SL) for accurate staging of all PDAC cases, except for unresectable cases with distant metastasis (UR‐M).

SL has evolved as a powerful tool for precise staging of cancer and continues to reflect the recent advances in laparoscopic surgery.^7^, ^8^ In PDAC, recent studies have reported that approximately 30% of patients present with radiologically negative occult distant metastases.^9^, ^10^ Previous reports also suggest that PDAC patients with occult distant metastases have a poor prognosis, and accurate staging by SL can reduce unnecessary noncurative resections. ^2^, ^11^, ^12^

Diagnostic and multidisciplinary treatment strategies for PDAC are constantly improving,^13^ resulting in the introduction of new surgical techniques.^14^ Systemic chemotherapies, such as FOLFIRINOX (fluorouracil, irinotecan, and oxaliplatin [FFX]) and gemcitabine along with nab‐paclitaxel (GnP), are generally used for unresectable PDAC.^15^, ^16^ Still, the optimal treatment strategy for PDAC with CY1 remains unclear. PDAC patients with CY1 are treated the same as UR‐M patients with systemic chemotherapy.^17^, ^18^ If multiple CY‐negative conversions were achieved after chemotherapy, the local tumor was resectable, and no new distant metastases appeared, a radical resection, so‐called conversion surgery, was performed.

Although there have been studies on the significance of SL in PDAC^9^ and the efficacy of systemic chemotherapy for PDAC with positive peritoneal dissemination or CY1,^19^ there have been no detailed studies on SL based on a resectability classification. SL is a useful tool for accurately diagnosing PDAC; however, it is not always possible to perform SLs in all PDAC patients due to the limitations of general anesthesia and surgical slots. Therefore, the establishment of indications for SL is needed. Otsuka et al. analyzed the results of peritoneal washing cytology in patients with resectable and borderline resectable PDAC who underwent either upfront surgery or radical surgery after neoadjuvant chemotherapy (NAC).^20^ They reported that pancreatic body or tail carcinoma was an independent risk factor for CY1 in the upfront group; however, they did not identify any significant factor in the NAC group, making it difficult to determine the indication for SL in NAC patients. However, their envisioned timing for SL was after NAC, which differs from our treatment strategy of performing SL at the time of diagnosis.

This is the first study to compare SL findings in PDAC patients before initial treatment and discuss SL indications and treatment strategies for CY1 cases based on the resectability classification.

## 2 METHODS

### 2.1 Study design and patients

We continuously collected clinicopathological information on SLs for PDAC patients at the University of Toyama (Toyama, Japan) and retrospectively reviewed 250 patients who underwent SLs before the initiation of treatment between August 2017 and January 2023. SLs were performed in all PDAC patients except UR‐M patients and classified PDAC patients who underwent SLs into three groups (resectable [R], borderline resectable [BR], and unresectable locally advanced [UR‐LA]) according to the National Comprehensive Cancer Network (NCCN) Guidelines (Version 2.2022).^21^ Patient radiological diagnoses were rigorously defined by several radiologists using thin‐slice multidetector row computed tomography (MDCT), three tesla gadolinium‐ethoxybenzyl‐diethylenetriamine pentaacetic acid‐enhanced magnetic resonance imaging (EOB‐MRI), and ^18^F‐fluorodeoxyglucose positron emission tomography (FDG‐PET). We performed MDCT, EOB‐MRI, and FDG‐PET on all PDAC patients before SL to exclude distant metastases and evaluate the resectability classification. Only cases that did not show any evidence of distant metastasis in all three radiological examinations were included in our study. The SL findings were evaluated for the presence of liver metastases (H factor), peritoneal dissemination (P factor), and peritoneal washing cytology (CY factor), with positive values defined as “1” and negative values as “0.” We examined the association between SL findings and clinicopathological factors and evaluated the results and treatment efficacy in CY1 cases based on the resectability classification. When SL in PDAC patients shows CY1, an abdominal port is inserted for future evaluation of peritoneal washing cytology, and systemic chemotherapy as for UR‐M is introduced.

Recently, the GnP regimen has been increasingly administered as systemic induction chemotherapy for patients with UR‐M PDAC in our department. After SL, at least three courses of systemic chemotherapy were administered to the relevant patients, and imaging evaluations were performed every 3 months. Peritoneal washing cytology evaluations and tumor marker measurements were performed monthly. The patients were scheduled for conversion surgery if the cytology showed negative conversion at least twice in a row from abdominal port sampling, the tumors were shrinking or unchanged, or tumor marker levels decreased. In principle, when peritoneal washing cytology collected from the abdominal port becomes negative multiple times, an SL is performed again before conversion surgery. For patients without abdominal ports, conversion surgery was scheduled after SL re‐examination and confirmation of CY‐negative conversion.

### 2.2 Staging laparoscopy in practice

We usually perform SL using a 3‐port technique with 5 mm trocars and 5 mm flexible scopes. After pneumoperitoneum at 10 mmHg, we performed peritoneal washing cytology and visual examination for liver metastases and peritoneal dissemination. Next, 100 mL of isotonic sodium chloride solution was injected into the pelvic floor and, as much as possible, was re‐collected and submitted as washed ascites for rapid pathological diagnosis. After centrifugation of the entire volume, the pathology department uses half of the cells obtained for rapid diagnosis and the other half for permanent diagnosis. The staining method was based on Papanicolaou staining, and Giemsa and Periodic Acid‐Schiff staining were used in combination. If peritoneal or hepatic nodules were found, they were resected simultaneously for diagnostic and therapeutic purposes. Resected nodules were also submitted for rapid diagnosis as necessary. Surgery was terminated if there were no non‐curative factors (H0P0CY0). If peritoneal dissemination or positive peritoneal washing cytology was detected intraoperatively, an abdominal port was inserted in the lower abdomen in all cases and was subsequently used to collect washing ascites fluid to evaluate the treatment efficacy.

### 2.3 Data picking up and clinicopathological evaluation

We collected data on PDAC patients based on the resectability classification from medical charts, including age, sex, tumor size, tumor location, and serum tumor markers, including carcinoembryonic antigen (CEA), serum carbohydrate antigen 19–9 (CA19‐9), duke pancreatic monoclonal antigen type 2 (DUPAN‐2), and carbohydrate antigen 125 (CA125) levels. Intraoperative factors such as H, P, and CY were also collected.

For H0P0CY1 cases, we also investigated whether postoperative systemic chemotherapy was introduced based on each resectability classification and regimen details. Systemic chemotherapy was administered to patients with maintained performance status, and most patients received GnP, while some patients received modified FFX, and some patients participated in clinical trials of S‐1 + intravenous and intraperitoneal paclitaxel (SP) or intravenous gemcitabine, intravenous nab‐paclitaxel, and intraperitoneal paclitaxel (GAP) administration.^19^, ^22^, ^23^ In addition, the subsequent CY‐negative conversion and conversion surgery rates were evaluated.

We also analyzed the association between clinicopathological characteristics and the H0P0CY1 in PDAC patients based on the resectability classification and SL findings. Our analysis of 115 patients with R‐PDAC has been published previously.^10^

### 2.4 Statistical analyses

Differences in the datasets between the groups were examined using the χ^2^ test. In addition, differences in quantitative variables were evaluated using the Student's *t*‐test or Mann–Whitney U test if the distribution was abnormal. We used univariate and multivariate logistic regression analyses to generate the odds ratios, including 95% confidence intervals, for clinical factors that would predict H0P0CY1 in each resectability classification. All optimal cutoff values for the logistic regression analyses were determined using the Youden index. Variables included in the multivariate models for H0P0CY1 had a *p*‐value of <0.05 in the univariate analysis. A *p* < 0.05 was defined as statistically significant. Statistical analyses were performed using JMP statistical software (version 16.0; SAS Institute).

## 3 RESULTS

### 3.1 Treatment course of PDAC patients with CY1 based on the resectability classification

The breakdown of the 250 cases by resectability classification was R:BR:UR‐LA = 131:48:71 cases (53:19:28%).

**FIGURE 1:**
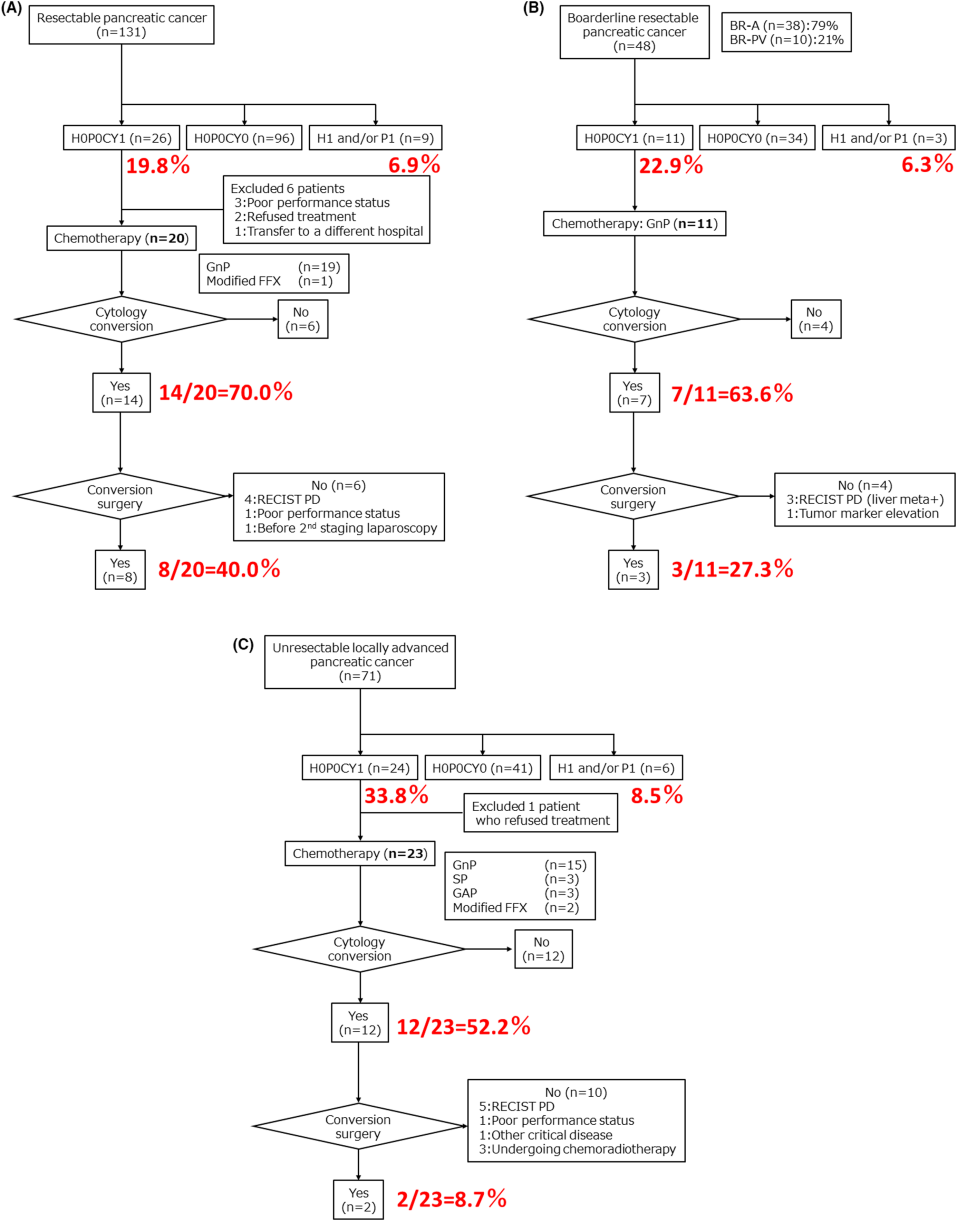
Treatment course of PDAC patients with positive peritoneal washing cytology based on resectability classification: (A) Resectable cohort (*n* = 131), (B) Borderline resectable cohort (*n* = 48), (C) Unresectable locally advanced cohort (*n* = 71). BR‐A, borderline resectable arterial system invasion; BR‐PV, borderline resectable portal venous system invasion; FFX, FOLFIRINOX (fluorouracil, irinotecan, and oxaliplatin); GAP, intravenous gemcitabine, intravenous nab‐paclitaxel, and intraperitoneal paclitaxel; GnP, gemcitabine along with nab‐paclitaxel; PD, progressive disease; RECIST, Response Evaluation Criteria in Solid Tumors; SP, S‐1 + intravenous and intraperitoneal paclitaxel.

Among the patients with resectable PDAC, 26 (19.8%) had H0P0CY1, and nine (6.9%) had H1 or P1. In the case of H0P0CY1, 20 patients received systemic chemotherapy (19 GnPs, one modified FFX), 14 (70.0%) achieved negative cytology conversion, and eight (40.0%) underwent conversion surgery (Figure 1A).

Among the patients with borderline resectable PDAC, 11 (22.9%) had H0P0CY1, and three (6.3%) had H1 or P1. All the BR patients with H0P0CY1 were treated with GnP, of which seven (63.6%) achieved negative cytology conversion. Conversion surgery was performed in three BR patients (27.3%) (Figure 1B).

Among the patients with unresectable PDAC, 24 (33.8%) had H0P0CY1, and six (8.5%) had H1 or P1. Regarding the 23 H0P0CY1 patients, excluding those who refused treatment, all patients received systemic chemotherapy (GnP in 15, SP in three, GAP in three, and modified FFX in two). Negative cytology conversion was achieved in 12 of the 23 patients (52.2%), and two (8.7%) underwent conversion surgery (Figure 1C).

The H0P0CY1 frequency increased proportionately to the degree of local progression (R:BR:UR‐LA = 20:23:34%). However, the frequencies of H1 or P1 were comparable (R:BR:UR‐LA = 6.9:6.3:8.5%). In most cases, systemic chemotherapy for H0P0CY1 was administered using a GnP regimen. The CY‐negative conversion rates were R:BR:UR‐LA = 70:64:52%, while conversion surgery rates were R:BR:UR‐LA = 40:27:9%. Both were inversely proportional to the degree of local progression.

### 3.2 Association between clinicopathological characteristics and the H0P0CY1 in PDAC patients by resectability classification

We calculated the cutoff values of each clinical factor for all three groups of the resectability classification and performed logistic regression analysis to examine risk factors for H0P0CY1. Table 1 shows the results for R pancreatic cancer. Univariate analysis showed that large tumor size and body or tail carcinoma were significantly associated with H0P0CY1. Multivariate analysis also showed that large tumor size and body or tail carcinoma were independent risk factors of H0P0CY1 (odds ratio, 4.38; 95% confidence interval, 1.61–11.92; *p* = 0.004, and odds ratio, 3.18; 95% confidence interval, 1.21–8.33; *p* = 0.019). The cutoff value for tumor size was 32 mm (Youden index 0.256). In BR patients, elevated DUPAN‐2 level was a significant factor in the univariate analysis (odds ratio, 38.50; 95% confidence interval, 1.02–1449.81; *p* = 0.049) (Table 2). In contrast, no significant factors were observed in the patients with UR‐LA (Table 3). For reference, Tables S1–S3 shows the results of the comparison regarding the presence of non‐curable factors in PDAC patients based on resectability classification.

**TABLE 1:** Logistic regression analysis for the predictive factors of H0P0CY1 in patients with resectable pancreatic cancer.

	*n*	Univariate analysis			Multivariate analysis		
		OR	95% CI	*p*‐value	OR	95% CI	*p*‐value
Age (years)							
≥65	104	3.91	0.41–37.69	0.239			
<65	18						
Sex							
Male	80	0.66	0.23–1.87	0.434			
Female	42						
Tumor size (mm)							
≥32	27	3.82	1.18–12.37	0.025*	4.38	1.61–11.92	0.004*
<32	95						
Location of the tumor							
Pbt	57	3.78	1.28–11.18	0.016*	3.18	1.21–8.33	0.019*
Ph	65						
CEA at diagnosis (ng/mL)^a^							
≥5.2	28	1.74	0.52–5.87	0.371			
<5.2	92						
CA19‐9 at diagnosis (U/mL)							
≥143	44	1.26	0.37–4.33	0.715			
<143	78						
DUPAN‐2 at diagnosis (U/mL)^a^							
≥200	52	0.96	0.28–3.28	0.954			
<200	69						
CA125 at diagnosis (U/mL)^a^							
≥14	40	1.02	0.32–3.30	0.971			
<14	60						

Abbreviations: CA125, carbohydrate antigen 125; CA19‐9, carbohydrate antigen 19–9; CEA, carcinoembryonic antigen; CI, confidence interval; DUPAN‐2, duke pancreatic monoclonal antigen type 2; OR, odds ratio; Pbt, pancreatic body and tail; Ph, pancreatic head.

^a^ Including missing value, *p < 0.05.

**TABLE 2:** Logistic regression analysis for the predictive factors of H0P0CY1 in patients with borderline resectable pancreatic cancer.

	*n*	Univariate analysis		
		OR	95% CI	*p*‐value
Age (years)				
≥72	26	31.60	0.67–1489.58	0.079
<72	19			
Sex				
Male	24	0.045	0.001–1.78	0.099
Female	21			
Tumor size (mm)				
≥30	21	14.29	0.70–292.53	0.084
<30	24			
Location of the tumor				
Pbt	13	0.18	0.01–4.21	0.289
Ph	32			
CEA at diagnosis (ng/mL)				
≥11.2	5	2.15	0.09–52.86	0.640
<11.2	40			
CA19‐9 at diagnosis (U/mL)				
≥581	9	8.34	0.27–261.50	0.227
<581	36			
DUPAN‐2 at diagnosis (U/mL)				
≥230	24	38.50	1.02–1449.81	0.049*
<230	21			
CA125 at diagnosis (U/mL)^a^				
≥15	23	1.58	0.15–16.70	0.704
<15	15			

Abbreviations: CA125, carbohydrate antigen 125; CA19‐9, carbohydrate antigen 19–9; CEA, carcinoembryonic antigen; CI, confidence interval; DUPAN‐2, duke pancreatic monoclonal antigen type 2; OR, odds ratio; Pbt, pancreatic body and tail; Ph, pancreatic head.

^a^ Including missing value, *p < 0.05.

**TABLE 3:** Logistic regression analysis for the predictive factors of H0P0CY1 in patients with unresectable locally advanced pancreatic cancer.

	*n*	Univariate analysis		
		OR	95% CI	*p*‐value
Age (years)				
≥76	13	3.51	0.70–17.67	0.128
<76	52			
Sex				
Male	34	0.37	0.10–1.41	0.146
Female	31			
Tumor size (mm)				
≥27	45	0.94	0.21–4.09	0.931
<27	20			
Location of the tumor				
Pbt	27	0.74	0.32–5.01	0.738
Ph	38			
CEA at diagnosis (ng/mL)^a^				
≥9.1	11	6.08	0.79–47.07	0.084
<9.1	53			
CA19‐9 at diagnosis (U/mL)				
≥1654	5	0.27	0.01–5.89	0.405
<1654	60			
DUPAN‐2 at diagnosis (U/mL)				
≥820	14	1.67	0.23–12.28	0.616
<820	51			
CA125 at diagnosis (U/mL)^a^				
≥39	7	0.60	0.07–5.43	0.651
<39	44			

Abbreviations: CA125, carbohydrate antigen 125; CA19‐9, carbohydrate antigen 19–9; CEA, carcinoembryonic antigen; CI, confidence interval; DUPAN‐2, duke pancreatic monoclonal antigen type 2; OR, odds ratio; Pbt, pancreatic body and tail; Ph, pancreatic head.

^a^ Including missing value.

### 3.3 Comparison of H0P0CY1 cases and CY‐negative conversion cases based on resectability classification

**FIGURE 2:**
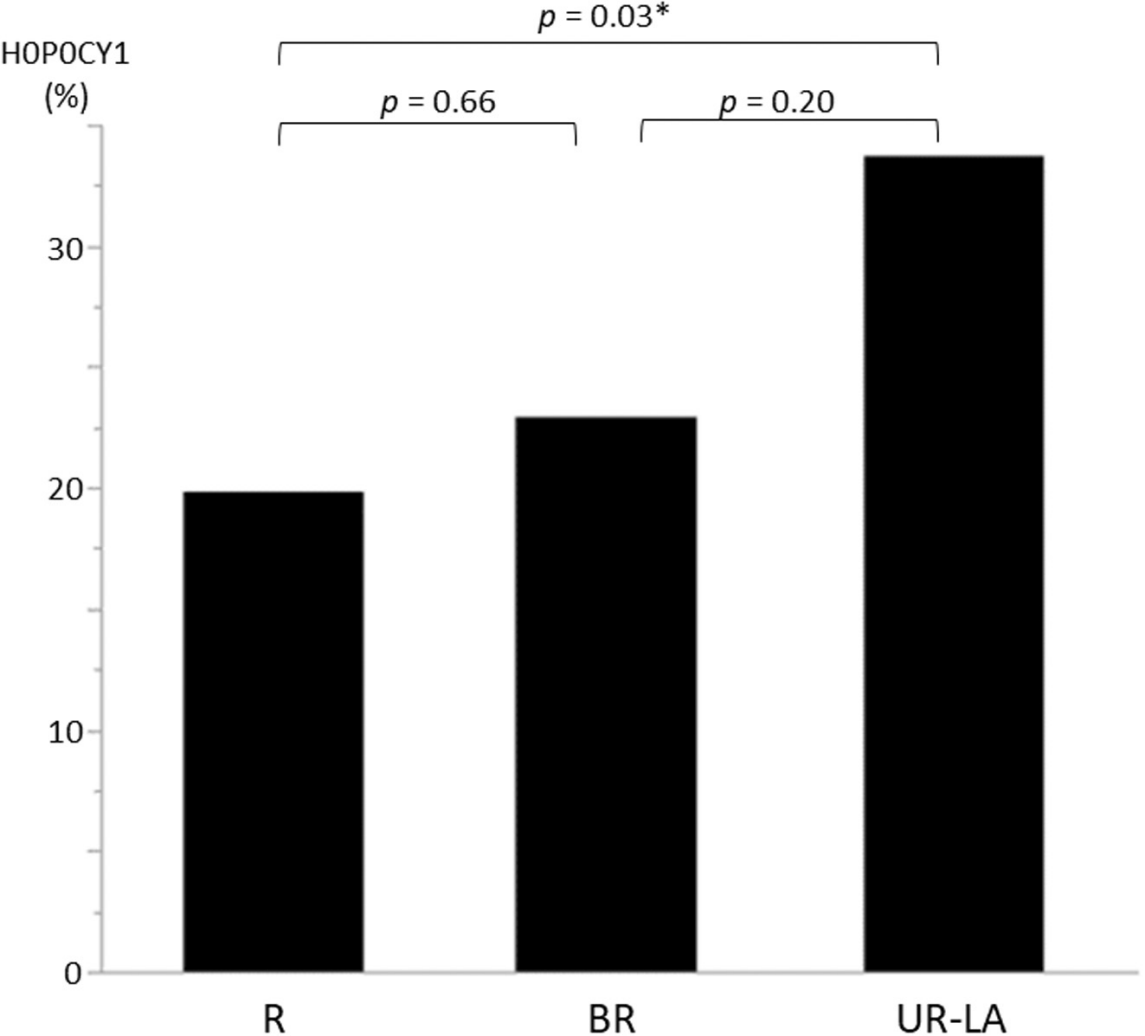
Comparison of H0P0CY1 cases based on resectability classification.

**FIGURE 3:**
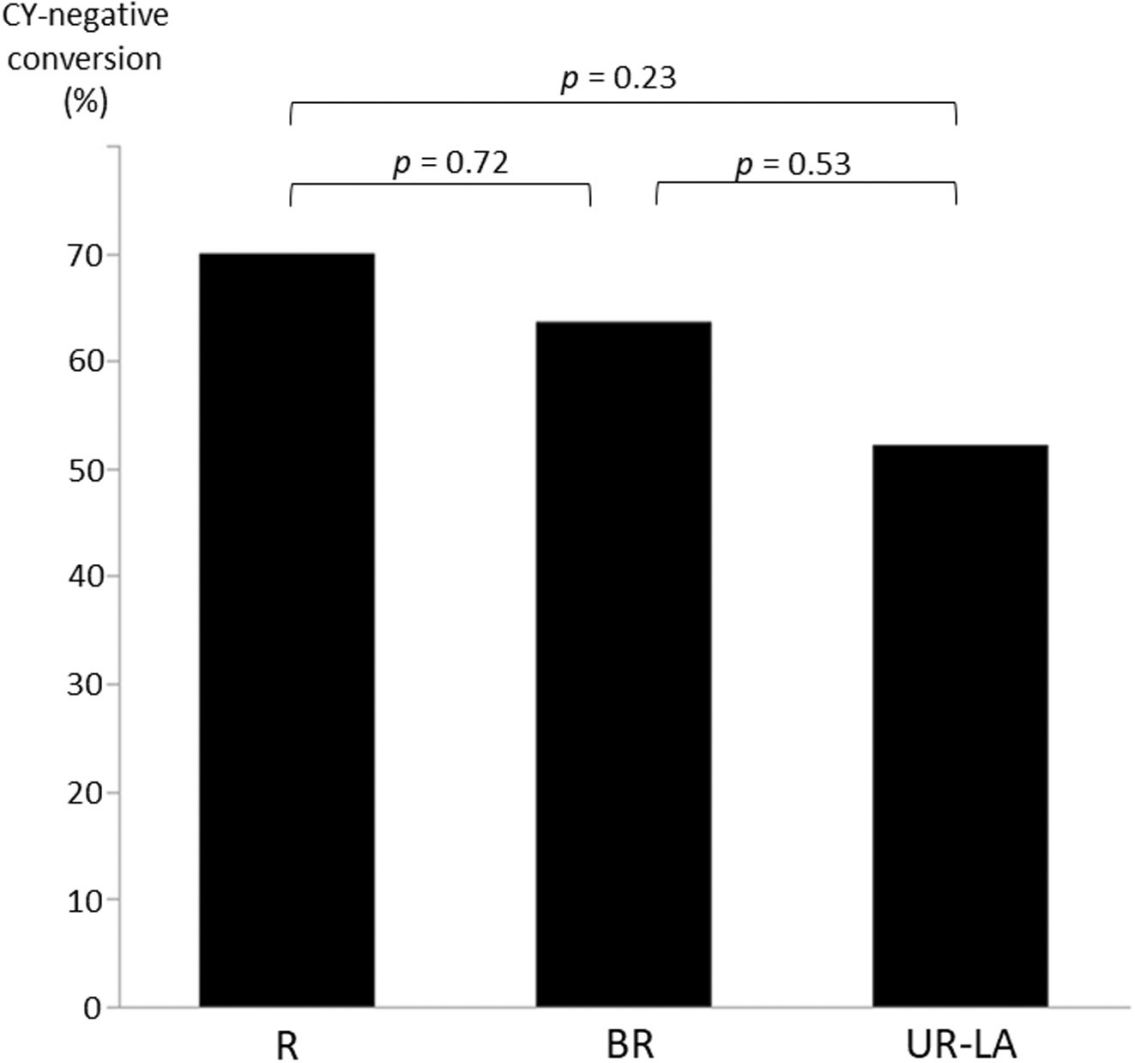
Comparison of CY‐negative conversion ratio based resectability classification.

Figure 2 shows the ratio of H0P0CY1 cases per resectability classification. The analysis revealed no significant differences between the R and BR (*p* = 0.66) and BR and UR‐LA (*p* = 0.20) cases; however, there was a significant difference between R and UR‐LA (*p* = 0.03) cases. Figure 3 shows the CY‐negative conversion ratio for each resectability classification. No significant differences were found between the classifications. The median (range) duration of chemotherapy until CY‐negative conversion was 2 (1–4) courses for the R group, 2 (1–4) courses for the BR group, and 2.5 (1–9) courses for the UR‐LA group.

## 4 DISCUSSION

With the advances in diagnostic imaging (MDCT, EOB‐MRI, FDG‐PET, and artificial intelligence) and diagnostic endoscopy (endoscopic ultrasound and endoscopic retrograde cholangiopancreatography), the diagnosis rate of pancreatic cancer is increasing.^24^, ^25^, ^26^ However, small distant metastases, such as small liver metastases or minimal peritoneal dissemination, are referred to as radiologically negative distant metastases, and they are difficult to diagnose early.^9^, ^27^ Takadate et al. reported that the detection rate of radiologically negative distant metastases in SLs was 48/146 (33%) in resectable PDAC.^9^ In our study, the detection rates of radiologically negative distant metastases in SLs were 34 (26%) in the R group, 14 (29%) in the BR group, and 30 (42%) in the UR‐LA group. The presence of approximately 30%–40% radiologically negative distant metastases is an important finding that cannot be ignored in PDAC treatment strategies. Even in patients with no evidence of distant metastases on MDCT, EOB‐MRI, and FDG‐PET, SL is a useful diagnostic tool for detecting radiologically negative distant metastases, and its use has increased in recent years for accurate staging.^18^, ^28^, ^29^, ^30^

Peritoneal washing cytology is widely used for diagnosing and staging multiple carcinomas, including gynecologic, gastric, and pancreatic cancers. Malignant cells can be identified in 7%–30% of peritoneal washing samples from patients with pancreatic cancer.^4^, ^31^, ^32^, ^33^ CY1 is a prognostic factor for poor prognosis in PDAC because it is considered to be a pre‐metastatic state and can cause a high rate of peritoneal recurrence even if radical surgery can be performed (CY1 vs. CY0 = median overall survival times: 17.5 vs. 29.4 months).^2^ The NCCN guidelines define CY1 as distant metastases (UR‐M).^21^ Recently, GnP and GS have been reported as chemotherapies for CY1 PDAC, and surgery for CY1 R‐PDAC after chemotherapy has been reported to have a better prognosis than upfront surgery.^34^ However, there is no established standard therapeutic strategy or consensus regarding CY1 PDAC. Our study showed that the CY‐negative conversion rate was lower in UR‐LA PDAC than in R or BR‐PDAC, and the conversion surgery rate after CY‐negative conversion also decreased as the disease progressed. Therefore, there is an urgent need for a regimen that combines efficacy and tolerability to enable conversion surgery in CY1 PDAC.

The criteria for conversion surgery of PDAC with CY1 remain unclear. Mitachi et al. considered a patient to be eligible for conversion surgery when all of the following criteria were met: CY‐negative conversion twice from abdominal port sampling, reduction in primary tumor size, decrease in maximum standardized uptake value on PET, no new lesions on imaging, and reduction in serum tumor marker levels.^35^ In addition, Satoi et al. reported maintenance of performance status after chemotherapy as a necessity for conversion surgery, and for UR‐LA PDAC patients, the prerequisites for conversion surgery are tumor shrinkage confirmed by contrast‐enhanced CT and decreased tumor marker levels, for CY1 patients CY negative conversion, and P0 by SL restudy if P1 by initial SL.^19^ Although there are no rigid rules regarding the chemotherapy sessions to be administered before conversion surgery, our previous study of UR‐LA PDAC showed no significant correlation between the chemotherapy duration and conversion surgery.^36^

In this study, 250 patients with PDAC who underwent initial SL were retrospectively reviewed based on the resectability classification to evaluate treatment outcomes for CY1 PDAC. Considering the current situation, wherein SL rates vary among institutions,^18^, ^37^ we also analyzed the predictors of H0P0CY1 for each resectability classification and evaluated SL indications to determine which patients should be considered for SL. Based on our analysis, patients with body or tail tumors and tumor size ≥32 mm for R‐PDAC, and elevated DUPAN‐2 (≥ 230 U/mL) for BR‐PDAC could be selected for SL. Tumor markers may be more associated with H and P factors compared to the CY factor. Sakaguchi et al. reported CA19‐9 ≥ 150 U/mL and tumor size ≥30 mm as “high‐risk markers” for radiologically negative occult distant metastasis in R and BR‐PDAC patients.^38^ Collectively, these results suggest that R and BR‐PDAC patients are a “high risk group” for harboring radiologically negative non‐curable factors, specifically if these individuals have tumors located in the body or tail of the pancreas, large tumor size, and elevated levels of CA19‐9 and DUPAN‐2 biomarkers. On the other hand, since no significant clinical factors to predict the presence or absence of non‐curative factors were found in UR‐LA PDAC, SL might be performed in all UR‐LA PDAC patients whenever possible.In this study, there were a certain number of patients who were judged RECIST PD and did not undergo conversion surgery even after achieving CY‐negative conversion (Figure 1A–C). This is because even if chemotherapy was effective against the disseminated cells, the effect may have been limited due to the high local tumor volume. CY1 cases have been shown to have a poorer prognosis than CY0 cases; therefore, it may be more acceptable to continue chemotherapy with the goal of achieving CY‐negative conversion.^2^, ^12^ To identify the optimal treatment strategy for CY1 PDAC, we designed and initiated a multicenter phase II study (WALCURE trial; jRCTs041230019).In the UR‐LA PDAC group, conversion surgery was feasible in 8.7% of H0P0CY1 cases (Figure 1C); in contrast, 14 of 41 (34.1%) H0P0CY0 patients were eligible for conversion surgery. In our previous report, we outlined the indication criteria for conversion surgery in our institution as follows: (a) tumor shrinkage or size unchanged; (b) no appearance of new metastatic sites; (c) performance status maintained at 0–1; (d) decrease in tumor marker values; and (e) technically resectable, i.e., “the SMA and tumor can be detached,” “hepatic artery invasion is suggested and requiring simultaneous resection, but reconstruction is possible,” or “portal vein concomitant resection is required, but reconstruction is possible.” Conversion surgery is considered when all of these criteria (a–e) are met.^36^

Despite the interesting findings of this study, our analysis had some limitations. Firstly, the retrospective single‐center nature of this study could have resulted in a selection bias. Secondly, the limited sample size may have influenced our results. Third, this analysis examined short‐term outcomes in terms of SL findings and conversion surgery rates and did not include a survival analysis of H0P0CY1 patients. We previously reported the results of a survival analysis based on SL findings in 115 R‐PDAC patients^10^; however, the observation period was insufficient. We plan to perform a reanalysis with a longer observation period. Therefore, further studies with a higher level of evidence, such as multicenter prospective studies, should be conducted in the future.

## 5 CONCLUSIONS

The CY1 rates, CY‐negative conversion rates, and conversion surgery rates for PDAC patients varied according to the degree of local progression. In the case of R and BR PDAC, SL could be considered in selected patients with pancreatic body or tail carcinoma, large tumor size, or high DUPAN‐2 level. However, in the case of UR‐LA PDAC, SL might be considered for all patients.

## AUTHOR CONTRIBUTIONS

Study concepts: Igarashi, Fukasawa, Watanabe, Fujii. Study design: Igarashi, Fukasawa, Watanabe, Fujii. Data acquisition: Igarashi, Fukasawa, Watanabe, Kimura, Itoh. Quality control of data and algorithms: Fujii. Data analysis and interpretation: Igarashi, Fukasawa, Watanabe, Kimura, Fujii. Statistical analysis: Igarashi, Fukasawa, Watanabe. Manuscript preparation: Igarashi. Manuscript editing: Tanaka, Shibuya, Yoshioka. Manuscript review: Hirabayashi, Fujii. All authors approved the final version of this manuscript.

## FUNDING INFORMATION

The authors have no sources of funding to declare.

## CONFLICT OF INTEREST STATEMENT

The authors have no conflicts of interest to declare for this study.

## ETHICAL APPROVAL AND CONSENT

The protocol for this research project has been approved by the Ethics Committee of the University of Toyama Medical Research Ethical Review Board, and it conforms to the provisions of the Declaration of Helsinki (Approval No. R2019138). Informed consent was obtained from all patients on an opt‐out basis. We complied with the Strengthening the Reporting of Observational Studies in Epidemiology (STROBE) guidelines.^39^

## Supporting information


Table S1.
Table S2.
Table S3.
