# Supplementary material for: Evaluating staging laparoscopy indications for pancreatic cancer based on resectability classification and treatment strategies for patients with positive peritoneal washing cytology
Source: Ann Gastroenterol Surg. 2023 Jul 18;8(1):124–32. doi: 10.1002/ags3.12719 (PMC10797817; doi:10.1002/ags3.12719)
Supplement: Supplementary file 1 — Table S1. Table S2. Table S3. [file AGS3-8-124-s001.docx]

**SUPPLEMENTARY TABLES**

**Supplementary Table 1.** Association between clinicopathological characteristics and presence of non-curable factors in patients with resectable pancreatic cancer

| **Characteristics** | **Patients without non-curable factors (H0P0CY0)**  **(n=96, 73%)** | **Patients with non-curable factors**  **(H1 or P1 or CY1)**  **(n=35, 27%)** | **p-value** |
| --- | --- | --- | --- |
|  | **Value** | **Value** |  |
| Age (years) | 72 (40–100) | 71 (54–84) | 0.65 |
| Sex |  |  | 0.16 |
| Male | 65 | 19 |  |
| Female | 31 | 16 |  |
| Tumor size (mm) | 22 (8–58) | 26 (11–64) | 0.083 |
| Location of the tumor |  |  | **0.014*** |
| Head | 56 | 12 |  |
| Body or tail | 40 | 23 |  |
| CEA at diagnosis (ng/mL) | 3.4 (1.0–89.3) | 3.7 (1.1–30.2) | 0.37 |
| CA19-9 at diagnosis (U/mL) | 72 (2–3,546) | 190 (1–9,473) | **0.043*** |
| DUPAN-2 at diagnosis (U/mL) | 96 (25–1,600) | 260 (25–1,600) | **0.027*** |
| CA125 at diagnosis (U/mL) | 12 (4–117) | 15 (5–396) | 0.40 |

*p < 0.05

Data are expressed as the median (range) or number of patients.

Abbreviations: CEA, carcinoembryonic antigen; CA19-9, carbohydrate antigen 19-9; DUPAN-2, duke pancreatic monoclonal antigen type 2; CA125, carbohydrate antigen 125.

**Supplementary Table 2.** Association between clinicopathological characteristics and presence of non-curable factors in patients with borderline resectable pancreatic cancer

| **Characteristics** | **Patients without non-curable factors (H0P0CY0)**  **(n=34, 71%)** | **Patients with non-curable factors**  **(H1 or P1 or CY1)**  **(n=14, 29%)** | **p-value** |
| --- | --- | --- | --- |
|  | **Value** | **Value** |  |
| Age (years) | 72 (45–82) | 76 (44–87) | 0.24 |
| Sex |  |  | 0.79 |
| Male | 18 | 8 |  |
| Female | 16 | 6 |  |
| Tumor size (mm) | 27 (15–180) | 31 (18–80) | 0.052 |
| Location of the tumor |  |  | 0.67 |
| Head | 24 | 9 |  |
| Body or tail | 10 | 5 |  |
| CEA at diagnosis (ng/mL) | 4.0 (0.7–31.6) | 4.5 (0.8–75.8) | 0.31 |
| CA19-9 at diagnosis (U/mL) | 88 (1–2,028) | 431 (2–2,586) | **0.048*** |
| DUPAN-2 at diagnosis (U/mL) | 172 (25–7,590) | 525 (25–2,480) | 0.22 |
| CA125 at diagnosis (U/mL) | 15 (5–124) | 20 (8–177) | **0.047*** |

*p < 0.05

Data are expressed as the median (range) or number of patients.

Abbreviations: CEA, carcinoembryonic antigen; CA19-9, carbohydrate antigen 19-9; DUPAN-2, duke pancreatic monoclonal antigen type 2; CA125, carbohydrate antigen 125.

**Supplementary Table 3.** Association between clinicopathological characteristics and the presence of non-curable factors in patients with unresectable locally advanced pancreatic cancer

| **Characteristics** | **Patients without non-curable factors (H0P0CY0)**  **(n=41, 58%)** | **Patients with non-curable factors**  **(H1 or P1 or CY1)**  **(n=30, 42%)** | **p-value** |
| --- | --- | --- | --- |
|  | **Value** | **Value** |  |
| Age (years) | 70 (40–84) | 71 (43–84) | 0.36 |
| Sex |  |  | 0.092 |
| Male | 26 | 13 |  |
| Female | 15 | 17 |  |
| Tumor size (mm) | 35 (15–75) | 32 (18–62) | 0.70 |
| Location of the tumor |  |  | 0.39 |
| Head | 26 | 16 |  |
| Body or tail | 15 | 14 |  |
| CEA at diagnosis (ng/mL) | 3.5 (0.8–35.4) | 4.9 (1.6–47.7) | 0.072 |
| CA19-9 at diagnosis (U/mL) | 117 (1–3,649) | 205 (1–20,131) | 0.11 |
| DUPAN-2 at diagnosis (U/mL) | 190 (25–1,600) | 127 (25–2,890) | 0.93 |
| CA125 at diagnosis (U/mL) | 15 (4–63) | 18 (7–111) | 0.30 |

*p < 0.05

Data are expressed as the median (range) or number of patients.

Abbreviations: CEA, carcinoembryonic antigen; CA19-9, carbohydrate antigen 19-9; DUPAN-2, duke pancreatic monoclonal antigen type 2; CA125, carbohydrate antigen 125.
